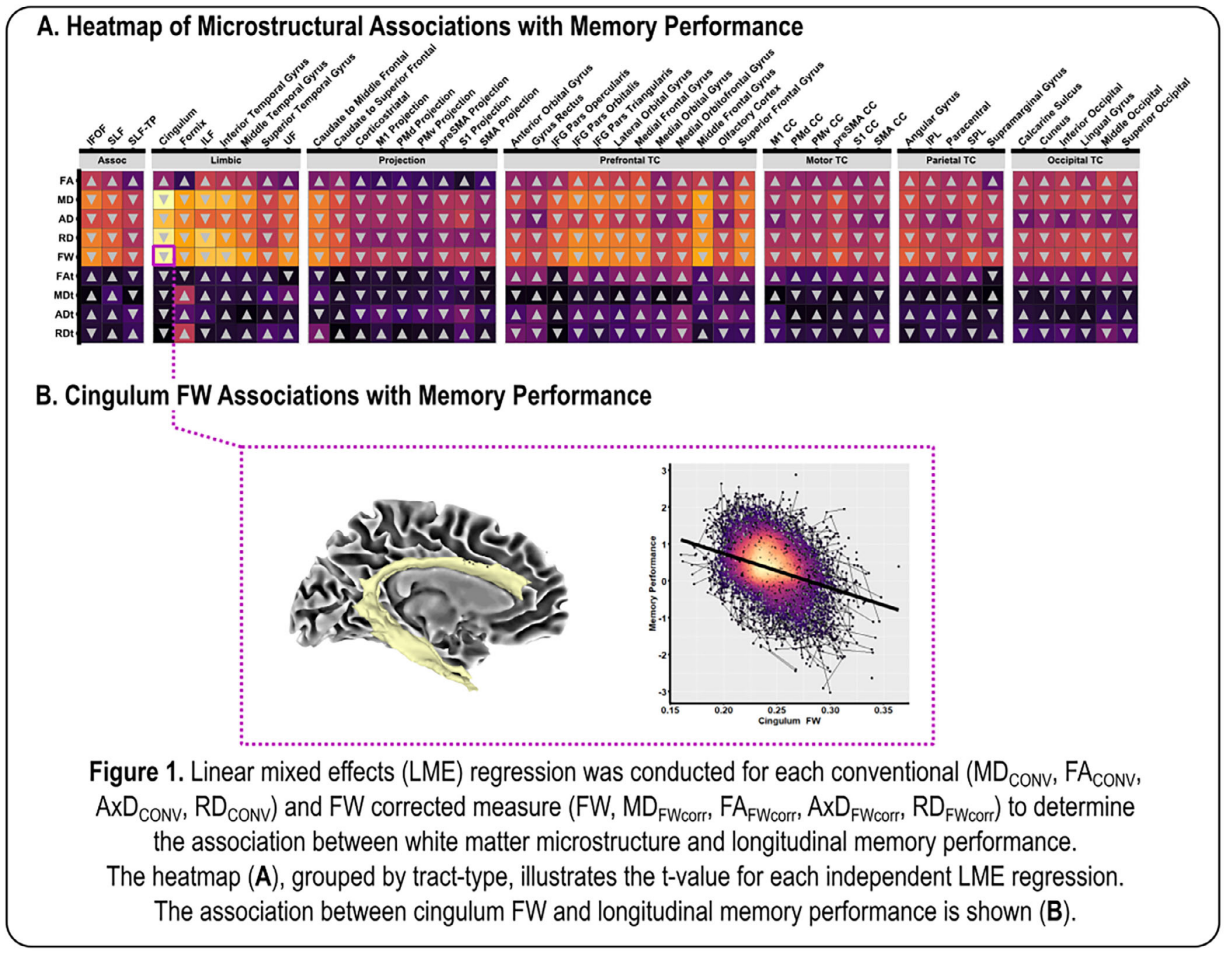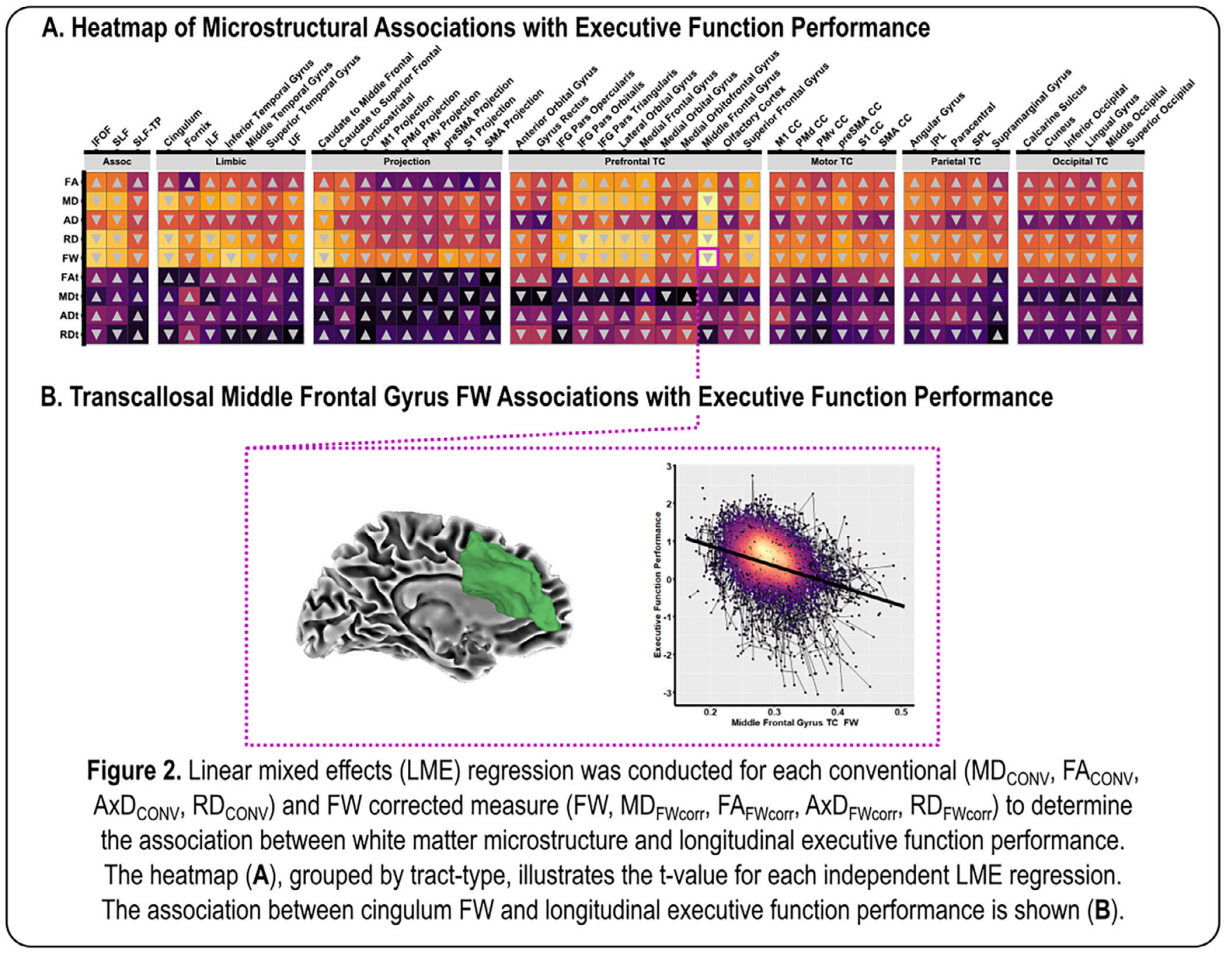# Uncovering the role of white matter microstructure in longitudinal memory and executive function decline: insights from a multi‐site study of 2,220 participants across 4,918 paired imaging‐cognition sessions

**DOI:** 10.1002/alz.090848

**Published:** 2025-01-09

**Authors:** Derek B. Archer, Chris Peter, Aditi Sathe, Yisu Yang, Alaina Durant, Niranjana Shashikumar, Kimberly R. Pechman, Logan C. Dumitrescu, Katherine A. Gifford, Shubhabrata Mukherjee, Brandon S Klinedinst, Michael L. Lee, Seo‐Eun Choi, Phoebe Scollard, Emily H. Trittschuh, Shannon L. Risacher, Lori L Beason‐Held, Yang An, Kurt Schilling, Bennett A. Landman, Julie A. Schneider, Lisa L. Barnes, David A. Bennett, Paul K. Crane, Walter A. Kukull, Sterling C. Johnson, Marilyn S. Albert, Angela L. Jefferson, Susan M. Resnick, Andrew J. Saykin, Timothy J. Hohman, Alzheimer's Disease Neuroimaging Initiative (ADNI)

**Affiliations:** ^1^ Department of Neurology, Vanderbilt University Medical Center, Nashville, TN USA; ^2^ Vanderbilt Memory and Alzheimer’s Center, Vanderbilt University Medical Center, Nashville, TN USA; ^3^ Vanderbilt Genetics Institute, Institute for Medicine and Public Health Vanderbilt University Medical Center, Nashville, TN USA; ^4^ Vanderbilt Memory & Alzheimer's Center, Vanderbilt University Medical Center, Nashville, TN USA; ^5^ Vanderbilt Memory & Alzheimer’s Center, Vanderbilt University Medical Center, Nashville, TN USA; ^6^ Vanderbilt Memory & Alzheimer's Center, Nashville, TN USA; ^7^ Vanderbilt Genetics Institute, Vanderbilt University Medical Center, Nashville, TN USA; ^8^ University of Washington, School of Medicine, Seattle, WA USA; ^9^ University of Washington, Seattle, WA USA; ^10^ Indiana University School of Medicine, Indianapolis, IN USA; ^11^ Center for Neuroimaging, Department of Radiology and Imaging Sciences, Indiana University School of Medicine, Indianapolis, IN USA; ^12^ National Institute on Aging, Baltimore, MD USA; ^13^ Department of Radiology & Radiological Sciences, Vanderbilt University Medical Center, Nashville, TN USA; ^14^ Vanderbilt University Institute of Imaging Science, Vanderbilt University Medical Center, Nashville, TN USA; ^15^ Department of Biomedical Engineering, Vanderbilt University, Nashville, TN USA; ^16^ Department of Electrical and Computer Engineering, Vanderbilt University, Nashville, TN USA; ^17^ Vanderbilt University Institute on Imaging Science, Vanderbilt University Medical Center, Nashville, TN USA; ^18^ Rush Alzheimer’s Disease Center, Rush University Medical Center, Chicago, IL USA; ^19^ Rush Alzheimer's Disease Center, Chicago, IL USA; ^20^ Rush University, Chicago, IL USA; ^21^ Department of Medicine, University of Washington, Seattle, WA USA; ^22^ Wisconsin Alzheimer's Institute, Madison, WI USA; ^23^ Alzheimer’s Disease Research Center, University of Wisconsin School of Medicine and Public Health, Madison, WI USA; ^24^ Wisconsin Alzheimer's Disease Research Center, Madison, WI USA; ^25^ Johns Hopkins University, Baltimore, MD USA; ^26^ Department of Medicine, Vanderbilt University Medical Center, Nashville, TN USA; ^27^ National Institute on Aging, National Institutes of Health, Baltimore, MD USA; ^28^ Indiana Alzheimer's Disease Research Center, Indianapolis, IN USA; ^29^ Indiana University, Indianapolis, IN USA

## Abstract

**Background:**

Recent research emphasizes the significance of white matter tracts and the free‐water (FW) component in understanding cognitive decline. The goal of this study is to conduct a large‐scale assessment on the role of white matter microstructure on longitudinal cognitive decline.

**Method:**

This study used a cohort collated from seven longitudinal cohorts of aging (ADNI, BIOCARD, BLSA, NACC, ROS/MAP/MARS, VMAP, and WRAP). In total, this dataset included 2,220 participants aged 50+ who had both diffusion MRI and harmonized composites of memory performance and executive function. This dataset included a total of 4,918 imaging sessions with corresponding cognitive data (mean number of visits per participant: 1.69 ± 1.67, interval range: 1‐10 years). Diffusion MRI was preprocessed using the PreQual pipeline and FW correction was used to create FW and FW‐corrected intracellular metrics. Conventional and FW‐corrected measures were harmonized using the Longitudinal ComBat package. Linear mixed effects regression was used for longitudinal analysis, in which we covaried for age, age squared, education, sex, race/ethnicity, diagnosis at baseline, APOE‐ε4 status, and APOE‐ε2 status. All models were corrected for multiple comparisons using the FDR approach.

**Result:**

For longitudinal memory performance, we found global associations with conventional diffusion MRI metrics, in which abnormalities were associated with lower memory performance. Following FW correction, we found that the FW metric itself was strongly associated with memory performance, in which higher FW was associated with lower memory performance and exacerbated decline. Interestingly, following FW‐correction the intracellular contributions were largely mitigated. As illustrated in Figure 1A, the most significant effects were found in the limbic tracts, with the most significant associations found for cingulum bundle FW (p=5.80x10^‐45^). Figure 1B illustrates the association between cingulum FW and longitudinal memory performance. Findings for longitudinal executive function performance are shown in Figure 2.

**Conclusion:**

To date, this is the largest study combining FW‐corrected diffusion MRI data and harmonized cognitive composites to understand cognitive trajectories in aging. Future studies evaluating how white matter microstructure may be incorporated into models of AD may further our knowledge into the neurodegenerative cascade of AD.